# CRESSENT: a bioinformatics toolkit to explore and improve ssDNA virus annotation

**DOI:** 10.1099/mgen.0.001632

**Published:** 2026-02-05

**Authors:** Ricardo R. Pavan, Matthew B. Sullivan, Michael J. Tisza

**Affiliations:** 1 Department of Microbiology, The Ohio State University, Columbus, Ohio, 43210, USA; 2 Centre of Microbiome Science, The Ohio State University, Columbus, Ohio, 43210, USA; 3 The Infectious Disease Institute, The Ohio State University, Columbus, Ohio, 43210, USA; 4 Department of Molecular Virology and Microbiology, The Alkek Center for Metagenomics and Microbiome Research, Baylor College of Medicine, Houston, TX, 77030, USA

**Keywords:** annotation, genomics, metagenomics, phylogenetic analysis, ssDNA, ssDNA virus

## Abstract

ssDNA viruses are important components of diverse ecosystems; however, it remains challenging to systematically identify and classify them. This is partly due to their broad host range and resulting genomic diversity, structure and rapid evolutionary rates. In addition, distinguishing genuine ssDNA genomes from contaminating sequences in metagenomic datasets (e.g. from commercial kits) has been an unresolved issue for years. Here, we present **CRESSENT** (**CRESS**-DNA **E**xtended a**N**notation **T**oolkit), a comprehensive and modular bioinformatic pipeline focused on ssDNA virus ‘genome-to-analysis’ and annotation. The pipeline integrates multiple functionalities organized into several modules: sequence dereplication, decontamination, phylogenetic analysis, motif discovery, stem-loop structure prediction and recombination detection. Each module can be used independently or in combination with others, allowing researchers to customize their analysis workflow. With this tool, researchers can comprehensively and systematically include ssDNA viruses in their viromics workflows and facilitate comparative genomic studies, which are often limited to dsDNA viruses, therefore leaving behind a crucial component of the microbiome community under study. Benchmarking analyses demonstrated that CRESSENT efficiently processes ssDNA virus datasets of varying scales, completing small family-level analyses within minutes and moderate comparative genomics studies within hours using standard computing resources. Its modular, parallelized design ensures scalability and low memory usage, making it accessible to research groups with diverse computational capacities.

**Impact Statement**

The study of ssDNA viruses, especially circular rep-encoding ssDNA (CRESS-DNA) viruses, has expanded rapidly with the rise of metagenomic sequencing, yet researchers face persistent challenges in their annotation and comparative analysis due to their compact genomes, overlapping reading frames and limited functional references. This work addresses a critical gap by enabling more comprehensive and standardized characterization of ssDNA viruses. The outputs presented here represent an incremental but impactful step toward advancing ssDNA virus research, offering solutions that will benefit virologists, microbial ecologists and evolutionary biologists working with viral dark matter. This work enhances the interpretability and comparability of ssDNA virus genomes, ultimately supporting efforts to map the ecology and evolution of one of the most understudied components of the global virosphere.

## Data Summary

CRESSENT is freely available online at https://github.com/ricrocha82/cressent. The documentation is available at https://cressent.readthedocs.io/en/latest/. Supplementary data are available at the Journal of General Virology online. The custom databases (contamination database and both family-level capsid and replication protein databases) are available for download from Zenodo under DOI: https://zenodo.org/records/15981951.

## Introduction

ssDNA viruses are among the most abundant and diverse biological entities on Earth, yet they remain one of the least understood groups of viruses [1,2]. The advent of metagenomic sequencing has led to a significant increase in the discovery of novel ssDNA viruses in diverse environments [2,4]. ssDNA viruses exhibit polyphyletic origins and have been repeatedly reshaped through gene exchange with plasmids, while the domain architectures of their Rep proteins vary markedly among bacterial, archaeal and eukaryotic hosts, collectively posing substantial challenges for comparative genomic analysis [5]. These challenges also include their small genome size (which can often lead to sequence removal in viromics workflows), rapid evolution rates, frequent recombination events and the presence of contaminating sequences in metagenomic datasets [6,7]. Furthermore, the absence of universally conserved genes further complicates taxonomic classification and comparative genomics of ssDNA viruses [8,9]. Due to these constraints, most studies examine a set of common genes within certain ssDNA groups, including Rep and Cap genes with conserved functions and structures [3,10, 11]. Yet, these genes can be extremely divergent at the sequence level, and recombination frequently occurs between the Rep and Cap genes and, in some cases, within the Rep gene itself. Therefore, identification of these events involves phylogenetic analysis of the Cap gene, the Rep gene and/or the nuclease and helicase domains of the Rep gene [5,12, 13], followed by tanglegrams to visualize recombination [13,14]. Short motifs (e.g. Walkers A and B) within the nuclease and helicase domains can vary by clade, and these are often visualized as sequence logos [13]. Non-coding stem-loops and iterons are additional conserved structures essential for the replication of these viruses and can also be used to further delineate ssDNA virus lineages [14,16].

**Fig. 1.**
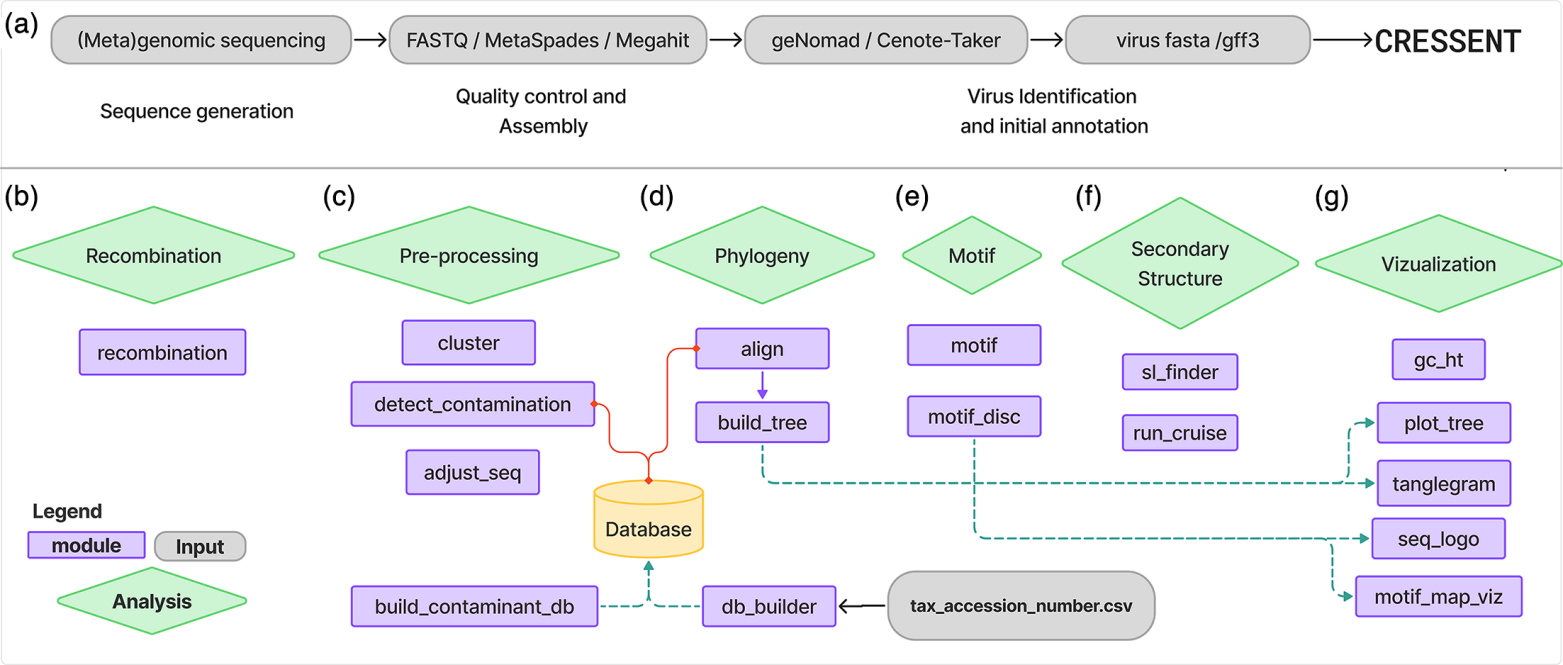
Flow chart depicting the CRESSENT modules (blue boxes), analysis (green diamonds) and processes (purple boxes). Grey and green dashed arrows indicate potential pipelines. (**a**) The analysis starts with the commonly used tools for viral contig discovery and annotation. Then, the user can decide which module to use: (**b**) recombination analysis; (**c**) dereplication, decontamination or adjustment of the sequence based on determined nucleotide or protein sequence; (**d**) phylogenetic analysis; (**e**) motif analysis; (f) secondary structure detection; (**g**) visualization such as phylogenetic tree, tanglegram, GC content heatmap, sequence logo or motif map visualization.

To address these challenges, we developed CRESSENT, a modular and comprehensive bioinformatic pipeline designed to analyse and annotate ssDNA virus sequences (Fig. 1). This tool consists of several modules, and each module can be used independently or in sequence, allowing researchers to customize their analysis workflow according to their specific research questions. Since the tool is modular, it allows for new modules to be added in the future. In this paper, we describe the functionality of each module and its integration into a cohesive analysis pipeline. With CRESSENT, researchers can enhance the efficiency and accuracy of ssDNA virus analysis and employ a standardized approach for comparative genomic studies of this important viral group.

The integration of multiple analysis methods provides researchers with a powerful toolkit for characterizing these important viral groups. The emphasis on data cleaning, visualization and flexible workflow design enhances the utility of CRESSENT for diverse research applications. We anticipate that this tool will contribute to a more standardized and reproducible approach to ssDNA virus analysis, facilitating comparative studies and advancing our understanding of viral diversity and evolution. Importantly, the modular architecture of this tool was specifically designed to facilitate continuous improvement and expansion. This extensibility ensures that the tool can evolve alongside the rapidly advancing field of viral metagenomics, providing a sustainable platform for ssDNA virus analysis for years to come.

## CRESSENT modules

Existing virus discovery pipelines such as GeNomad [17], VirSorter2 [18] and Cenote-Taker3 [4] are highly effective at identifying viral contigs but offer limited post-processing capabilities specifically tailored to ssDNA viruses. CRESSENT addresses this gap by integrating widely used and validated bioinformatic tools into a single, modular and reproducible framework optimized for the evolutionary characteristics of ssDNA viruses. This design eliminates the need for users to manually locate, install and configure disparate tools, saving substantial time and ensuring that commonly required analyses in ssDNA viral genomics are performed in a consistent and standardized manner. The primary function of CRESSENT is therefore to serve as an auxiliary toolkit that refines the annotation and analysis of putative ssDNA viral sequences identified by upstream virus discovery programmes [4,17, 18]. Moreover, its planned integration into the iVirus cyberinfrastructure [19] will facilitate seamless interoperability with other virus identification and annotation workflows.

The modular design of CRESSENT allows flexible integration of its components depending on specific research questions and dataset characteristics. Using the output of virus discovery tools as input (i.e. FASTA and GFF files), a typical workflow might begin with dereplication to reduce dataset complexity, followed by decontamination to remove potential laboratory contaminants. The resulting non-redundant and clean sequences can then undergo recombination detection, phylogenetic analysis and motif discovery, with stem-loop and iteron annotation further revealing replication-associated structures.

Although users may conceptually follow this logical sequence, CRESSENT does not enforce a fixed order of execution. Most modules are designed to accept standard FASTA or GFF3 files not only from other CRESSENT modules but also from external tools, allowing users to enter the workflow at any stage according to their data type or analytical goals. Even within a specific workflow such as phylogenetic reconstruction, iterative refinement is often necessary, as optimal alignment and trimming parameters can vary substantially between ssDNA viral families due to differences in genome organization and protein conservation.

The tool is written primarily in Python and R and is operated through a command-line interface. Each of the six modules is described in detail below.

## Recombination detection

Recombination is a major driver of genetic diversity and evolution in ssDNA viruses [20,21]. The accurate detection of recombination events is therefore crucial for understanding viral evolution, taxonomy and epidemiology. The recombination module allows users to run specific or all methods for recombination detection and provides options for customization through a configuration file (Fig. 1b).

This module identifies recombination in nucleotide sequences using a suite of methods integrated within the OpenRDP (Recombination Detection Program) framework. The analysis incorporates multiple statistical and phylogenetic approaches to improve detection sensitivity and reliability [22]. The RDP method examines sequence triplets for recombination signals using a recursive segmentation algorithm, while 3Seq detects recombination based on phylogenetic incongruence among three sequences. GENECONV identifies gene conversion events by scanning for unusually long identical fragments shared between sequences. MaxChi and Chimaera both apply chi-square tests to detect breakpoints by comparing observed and expected substitutions across aligned sequences, with Chimaera using a more refined partitioning strategy. Bootscan assesses phylogenetic relationships along the sequence alignment by generating bootstrapped trees for sliding windows, highlighting regions of differing ancestry. SiScan extends this approach by calculating similarity scores across windows to detect potential recombination breakpoints. The methods implemented in this tool have been widely used in studies of viral recombination, including applications to circoviruses and parvoviruses, demonstrating their effectiveness across diverse ssDNA virus groups [23,24].

The module accepts multiple-sequence nucleotide alignments (FASTA format) and produces a tab-delimited CSV table summarizing each detected event, including breakpoints, parental sequences, test statistics and the methods supporting it. Outputs also include timestamped log files documenting execution parameters and software versions to ensure reproducibility.

CRESSENT’s recombination module uses the OpenRDP configuration, balancing detection sensitivity with runtime efficiency. Circular genome scanning is disabled by default (circular_genome=False) to prevent false breakpoints at contig termini, and Bonferroni correction ensures conservative significance control across multiple pairwise comparisons. Analytical *P*-values (num_permutations=0) accelerate analyses of large alignments, while events are reported when detected by at least one method (min_num_detecting_events=1). Each recombination algorithm operates with parameter sets tuned to compact, highly divergent ssDNA genomes: RDP scans 30-nt windows, GENECONV permits short identical tracts and treats indels as polymorphisms and Bootscan employs 200 bp sliding windows (20 bp steps, 100 bootstraps) under a Jukes–Cantor model with ≥70% bootstrap support.

Complementary chi-square methods (MaxChi, Chimaera) use windows of 100–200 bp and require at least 60–70 variable sites per test, while SiScan performs similarity profiling with equivalent window sizes and permutation-based *P*-value estimation (1,100 permutations). These settings prioritize reproducibility and minimize false positives without oversmoothing short viral genomes. All parameters can be adjusted through an .ini file, enabling users to tighten detection criteria for high-confidence evolutionary analyses or to relax them for exploratory scanning across diverse ssDNA virus lineages.

## Data pre-processing

The pre-processing step (Fig. 1c) involves three modules to cluster, clean or adjust the sequence for further analysis. First, the decontamination module addresses a critical issue in metagenomic studies: the presence of laboratory contaminants or ‘kitome’ sequences [25,27]. The user has full flexibility to define how stringent the decontamination process should be. Second, the user can use the adjust_seq module to rearrange the sequence, for example, to begin with conserved nonanucleotide sequences.

## Dereplication

The third method in data pre-processing, sequence clustering and dereplication, is widely used in viral metagenomics to manage dataset complexity and avoid bias from overrepresented sequences [7]. CRESSENT adheres to the current community standard for dereplication of viral contigs, as outlined in the MIUViG guidelines: a 95% ANI over at least 85% of the contig length (AF) [7]. This module utilizes a combination of tools, including anicalc.py and aniclust.py from CheckV [28] and blast [29], to calculate pairwise ANI and cluster sequences based on user-defined similarity thresholds. It outputs a vOTU catalogue with a designated cluster representative, the full set of clustered sequences, pairwise ANI values and supporting blast alignments. By removing redundancy while preserving true biological diversity, the module markedly reduces computational load for all downstream analyses.

## Decontamination module and contamination database

Laboratory contamination is a concern in microbiome metagenomic studies [25,26, 30] and can lead to the misidentification of microbial taxa, inflate diversity estimates or result in erroneous biological interpretations if not properly controlled for through rigorous contamination-aware protocols. The systematic identification and removal of contaminant sequences is therefore essential for accurate analysis and interpretation of results.

It has been demonstrated that commercial reagents and extraction kits can contain viral DNA that may be misinterpreted as novel findings [31]. To mitigate this, a comprehensive database with 510 potential viral contaminants in laboratory reagents has been compiled [32]. Contaminants included four small circular virus-like genomes [25]. CRESS-like viruses were also found in laboratory reagents and may be constituents of the ‘kitome’. For instance, the presence of CRESS-like viruses such as Parvovirus-like Hybrid Virus [33] and Rengasvirus [26] in negative control samples can be mistaken for novel or biologically relevant viruses, which highlights the need to cross-reference against known reagent-associated sequences to avoid erroneous conclusions in virome and pathogen discovery research.

To limit contamination, this module uses blast to compare input sequences against a curated database of known contaminants derived from five published studies [25,26, 31,33]. The module produces decontaminated sequences, decontamination statistics and blast results for tracking potential contaminants. To balance the removal of contaminants while retaining genuine sequences, users can fine-tune the blast parameters, specifically the e-value (default: 1e-10), percent identity (default: 90) and alignment coverage (default: 50).

## Phylogenetic analysis, tree visualization and Cap and Rep databases

The phylogenetic analysis module (Fig. 1d) comprises three main components: sequence alignment, phylogenetic tree construction and tree visualization/annotation. Our implementation also includes user-friendly customization options, such as selecting family-level protein sequences from a ICTV-derived database focused on replication-associated (Rep) and capsid (Cap) proteins.

The alignment component uses MAFFT [34] for multiple sequence alignment and trimAl [35] for alignment trimming, while the tree construction component utilizes IQ-TREE2 for maximum likelihood phylogenetic inference [36]. MAFFT and trimAl have been used for viral sequence alignment due to their accuracy and efficiency, which is particularly important for divergent viral sequences [3,8, 14]. IQ-TREE2 represents the state-of-the-art in maximum likelihood phylogenetic inference and has been used in viral phylogenomics [5]. Similar approaches have been used to investigate the evolutionary history of CRESS-DNA viruses, as well as to examine the diversity and evolution of ssDNA viruses in avian hosts [37,38]. The visualization of phylogenetic trees leverages specialized R packages, including ggtree [39], ape [40] and dendextend [41]. Additionally, when the tanglegram module is activated, the Robinson–Foulds distance is automatically calculated to quantify the topological dissimilarity between two phylogenetic trees. This metric provides a standardized way to assess how similar or different two trees are, which is essential for comparing phylogenetic reconstructions and evaluating the impact of different analytical methods or datasets [42].

In addition, a taxonomy-based database builder module is available for specific taxonomic levels (Fig. S1, Supplementary Material 1, available in the online Supplementary Material). The database is derived from the ICTV-recognized Master Species List (MSL) (MSL39.v4; https://ictv.global/sites/default/files/MSL/ICTV_Master_Species_List_2023_MSL39.v4.xlsx). The tool also supports user-generated databases and provides two dedicated modules for phylogenetic tree visualization: one for standard tree visualization and another for tanglegram plotting (Fig. 1g).

## Motif analysis and visualization

Motif analysis is crucial for identifying functional elements in viral genomes, such as replication origins (ori), protein binding sites and conserved protein domains. In ssDNA viruses, conserved motifs within replication-associated proteins are especially informative, as they often reflect evolutionary constraints and mechanistic roles in rolling-circle replication. For example, motifs such as the HUH endonuclease domain and the superfamily 3 helicase domain are required for site-specific DNA cleavage and unwinding of the DNA strand, respectively. These functional domains are highly conserved across diverse ssDNA viruses, indicating strong purifying selection. As a result, their conservation across viral families can provide clues to the biochemical mechanisms of replication, robust markers for evolutionary comparisons and taxonomic classification [1,3, 8, 43, 44].

The MOTIF modules (Fig. 1e) enable both pattern-based motif searching and *de novo* motif discovery in ssDNA viral sequences. The input can be nucleotide or protein sequences as FASTA files. Both modules produce several outputs, including motif positions, sequence logos of reference and user input motifs and genome maps to visualize the distribution of identified motifs.

For *de novo* motif discovery, the module integrates MEME for motif identification and optionally ScanProsite for scanning against known protein motifs. Both tools have been used for *de novo* motif discovery in viral genomics [45,47]. For example, tools such as MEME have been employed to explore conserved motifs in CRESS-DNA viruses, offering insights into their evolutionary relationships and classification [48,49]. Similarly, motif identification platforms like ScanProsite have been applied to viral metagenomic sequences from avian hosts to detect functionally relevant sequence patterns [50]. The pattern-based searching functionality in our tool is particularly useful for identifying known functional motifs, such as the Walker A and Walker B motifs in Rep proteins, which are indicators of rolling-circle replication mechanisms common in ssDNA viruses [8,44, 51]. For pattern-based searching, the module uses regex and the seqkit tool [52] to identify user-defined patterns and optionally split sequences at motif occurrences.

## Putative stem-loop and iteron annotation

DNA secondary structures typically form the origin of replication and serve as recognition sites for viral Rep proteins [14,15, 53]. Stem-loop structures and iterons are essential for viral replication in many ssDNA viruses. As a well-studied example, the stem-loop structure in Faba bean necrotic yellows virus functions as the origin of replication, with a conserved nonanucleotide sequence within the loop being essential for initiating rolling circle replication [54]. Additionally, a set of iterative sequences (iterons) serves as specific binding sites for Rep proteins, and their spatial arrangement plays a crucial role in determining replication efficiency in *Geminiviridae*. These structures represent critical functional elements that are conserved across diverse ssDNA viral families despite high sequence variability in other genomic regions [55].

This module identifies and annotates critical noncoding secondary structures in ssDNA viral genomes: stem-loops and iterons (Fig. 1f). CRESSENT includes two modules for these analyses: StemLoop-Finder for identifying DNA hairpin structures and CRUISE (CRiteria-based Uncovering of Iteron SEquences) for detecting iteron repeats that serve as recognition sites for replication proteins [56,57]. StemLoop-Finder uses the ViennaRNA library for predicting secondary structures and scoring potential stem-loops based on deviation from ideal stem and loop lengths. CRUISE identifies iteron sequences, which are typically found near the origin of replication in many ssDNA viruses.

## Visualization and outputs

Visualization components are integrated throughout the pipeline (Figs 1g and S2–S4 Supplementary Material 1), including tree visualization with ggtree, and sequence logo generation for motif representation. The visualization tools make use of established R packages such as ggtree [39], ggtreeextra [58] and ggseqlogo [59], providing publication-quality figures for downstream use. The output files from each module are designed to be compatible with the input requirements of subsequent modules, facilitating seamless integration. Also, they can be used as inputs for other tools not included in CRESSENT.

## Benchmarking

To assess the computational performance and scalability of CRESSENT, we benchmarked its core modules using new metagenome-assembled genomes from two representative CRESS-DNA virus families: a small *Naryaviridae* dataset [60] containing three genomes (six proteins) and a *Genomoviridae* dataset [61] comprising 32 genomes (98 proteins). For each family, we independently evaluated analyses of the capsid (Cap) and replication-associated (Rep) proteins across four core computational modules: sequence clustering, multiple sequence alignment, phylogenetic tree inference and *de novo* motif discovery and annotation. All tests were conducted on a high-performance computing cluster (Red Hat Enterprise Linux 9.4) equipped with 40-core Intel Xeon processors and 187–375 GB RAM, using 40 threads for parallel execution.

The *Naryaviridae* dataset illustrated the pipeline’s efficiency for small-scale analyses. Cap protein processing completed rapidly: clustering in 9 s (69 MB), alignment in <2 s (70 MB), motif discovery in 19 s (71 MB) and tree construction in 2.7 min (69 MB). Rep protein analyses showed similar performance: clustering (2 s), alignment (2 s), motif discovery (19 s), and tree construction (2.3 min) with all steps finishing within 3 min and <75 MB memory usage. For the *Genomoviridae* dataset, Cap protein clustering was completed in <3 s (87 MB), alignment required 11.4 min (988 MB), motif discovery 33 s (87 MB) and tree construction 5 h 24 min (1.37 GB). Rep protein analyses showed comparable clustering (6 s, 85 MB) and alignment (11.8 min, 1.44 GB) times, whereas tree inference was substantially more demanding (19 h 25 min, 1.96 GB), reflecting the higher sequence diversity and phylogenetic complexity of Rep proteins.

These results demonstrate that CRESSENT efficiently handles datasets ranging from small-scale studies to moderate-sized comparative genomics projects with reasonable computational requirements. Parallel implementation enables effective use of multicore systems, with high central processing unit (i.e. CPU) utilization observed during alignment and phylogenetic inference. Memory demands remain modest for most modules (typically 70–90 MB for clustering, alignment and motif discovery), with tree building constituting the primary computational bottleneck (70 MB–2 GB, depending on dataset size and protein diversity). Consequently, complete analyses can be executed on standard laboratory workstations for small datasets or scaled efficiently on high-performance computing platforms for larger projects, making CRESSENT accessible to laboratories with varied computational capacity.

Importantly, CRESSENT is designed for targeted analyses at the family, genus or species level, where phylogenetic signals remain informative for comparative genomics. Given the extensive sequence divergence among ssDNA viruses even within single genera [5,13], joint analyses of multiple families or highly divergent lineages would markedly increase runtime and memory requirements, particularly during alignment and tree inference, while potentially reducing the biological interpretability of results due to alignment ambiguity and phylogenetic saturation (Fig. S1B–E).

## Supplementary material

10.1099/mgen.0.001632 Supplementary Material 1.

## Abbreviations

CRESSENT: CRESS-DNA Extended aNnotation Toolkit
CRUISE: CRiteria-based Uncovering of Iteron SEquences
MSL: Master Species List
vOTUs: viral operational taxonomic units
