# Supplementary material for: CRESSENT: a bioinformatics toolkit to explore and improve ssDNA virus annotation
Source: Microb Genom. 2026 Feb 5;12(2):001632. doi: 10.1099/mgen.0.001632 (PMC12877143; doi:10.1099/mgen.0.001632)
Supplement: Supplementary Material 1. [file mgen-12-01632-s001.pdf]

Supplementary Materials for

CRESSENT: a Bioinformatic Toolkit to Explore and Improve ssDNA Virus Annotation

Pavan, RR; Sullivan, MB; Tisza, MJ

S1. CRESSENT MODULES

S1.1 Recombination Detection

Under the hood, CRESSENT automatically manages and validates the required binaries for 3Seq and GENECONV, compiling them from source if precompiled versions are incompatible with the user's platform. This automation ensures seamless use across Linux, macOS, and Windows systems. The recombination module is thus designed for rapid, reproducible screening of aligned ssDNA viral genomes, guiding users in refining phylogenetic datasets, identifying recombinant domains, and improving the accuracy of downstream evolutionary analyses.

S1.2 Decontamination

This module removes likely "kitome"/reagent contaminants by (i) building a curated contaminant database from an accession list and (ii) screening user sequences against that database. The builder (build_contamination_db.py) fetches nucleotide records (and their CDS-encoded proteins) from NCBI Entrez via Biopython, writes FASTA(s) plus per-record metadata (TSV), and logs all actions (batching and retries to be polite to NCBI). The screener (detect_contamination.py) auto-detects nucleotide vs protein input, constructs a BLAST database with makeblastdb, and runs BLAST+ (blastn for nucleotides, blastp for proteins) to flag matches to contaminants; it then writes a filtered FASTA, a statistics report (counts and contamination rate), optional BLAST hits (TSV), and a log.

Dependencies (underlined here in text): Biopython/Entrez and pandas for I/O and metadata; BLAST+ (blastn, blastp, makeblastdb) as the alignment engine; standard Python logging. Inputs: (a) user sequences in FASTA; (b) a contaminant FASTA built from a CSV of accessions (or a prebuilt FASTA). Outputs: filtered FASTA, stats TXT, optional BLAST TSV, logs, and (from the builder) nucleotide/protein FASTAs plus metadata TSVs. Use this module immediately after discovery/dereplication to prevent downstream phylogeny/motif analyses from being driven by reagent-derived sequences.

Screening thresholds default to evalue=1e-10, identity=90%, and query coverage=50% (format 6 with qcovs), which are conservative enough to catch known reagent sequences while minimizing over-filtering of true ssDNA contigs; increase to 95–98% identity and ≥70% coverage for high-stringency curation or relax for exploratory scans. Threads are configurable (-t/--threads). Identity filtering is passed directly to blastn (-perc_identity) and applied post-hoc for both programs when parsing; coverage is enforced at parse time. Sequence type is auto-detected from the alphabet but can be forced via --seq-type {nucl,prot}. The BLAST DB is

created on the fly if missing, ensuring portability. For database construction, the builder accepts --batch-size (default 10) and --email (Entrez identification), downloads nucleotide plus CDS-protein sequences, annotates descriptions as [Contaminant], and emits both FASTA(s) and metadata TSV(s). All runs are fully logged (paths, parameters, versions) to support reproducibility and reviewer audit.

S1.3 Phylogenetic Analysis

CRESSENT's phylogenetic module implements a reproducible workflow for ssDNA viral proteins comprising multiple-sequence alignment, objective trimming, model-aware tree inference, and publication-quality visualization. Protein sequences (Rep/Cap, optionally merged with curated family references) are aligned with MAFFT (--localpair --maxiterate 1000 --leavegappyregion, tunable --ep, multithreaded) to accommodate short, divergent domains; low-information sites are then filtered with trimAl (-gt, default 0.2) to reduce noise while retaining phylogenetic signal.

Trees are inferred with IQ-TREE 2, using ModelFinder (-m MFP) for automatic substitution-model selection and ultrafast bootstrap support (-B, default 1000; -T AUTO), with pass-through of additional arguments as needed. The pipeline records sanitized tip labels and metadata, and renders figures via an R wrapper using treeio/ggtree/ggtreeExtra/ape/Biostrings, supporting rectangular, circular, or unrooted layouts, optional alignment panels, tip grouping and coloring, and user-defined geometry (offsets, open angles, width/height). Inputs include FASTA alignments and optional sample metadata; outputs comprise aligned and trimmed FASTAs, IQ-TREE logs and treefiles, a name-mapping table, and a vector PDF figure.

Default parameters (MAFFT ep=0.123, trimAl gap threshold 0.2, IQ-TREE MFP with 1000 bootstraps) are chosen to balance sensitivity and specificity for compact, highly divergent ssDNA proteins, yielding robust, manuscript-ready phylogenies.

S1.4 MOTIF Analysis

CRESSENT's motif module couples *de novo* discovery with targeted scanning and visualization to characterize short, conserved signatures in ssDNA viral proteins and genomes. For *de novo* inference, we call MEME (via motif_disc.py) with sequence-type auto-detection and user-exposed controls on motif count and width (-nmotifs, -minw, -maxw, plus --meme_extra pass-through). MEME outputs (meme.xml, EPS logos) are parsed with Biopython (Bio.motifs) to produce two machine-readable tables: a consensus summary (consensus_table.csv) and an instance-level table (motif_table.csv) that records, per sequence, the matched subsequence, coordinates, strand, and the regular-expression form extracted from MEME. As a complementary, knowledge-driven layer, users can optionally annotate proteins against curated signatures by enabling ScanProsite (Biopython ExPASy/ScanProsite), which yields a tab-separated report (scanprosite_results.csv) containing accessions, start/stop positions, and retrieved textual annotations. This pairing (MEME for discovery, Prosite for curation) is intended to capture both novel Rep/Cap signatures typical of compact, rapidly evolving ssDNA proteins and established catalytic or binding motifs.

For targeted motif mapping and figure generation, motif.py employs SeqKit to locate user-specified patterns (--pattern accepts literal or regex), with options to remove alignment gaps beforehand (--remove-gaps) and to split sequences at the first hit for downstream domain analyses (--split-sequences). Outputs include a coordinates table (pattern_positions.txt) and, when requested, publication-quality sequence logos rendered by an R helper (seq_logo.R) with tunable geometry (--width/--height), titling, and grouped panels (--split-logo with --metadata, --group-label, --ncol).

To summarize motif architecture across proteins, motif_map_viz.py standardizes either MEME tables or Prosite outputs and produces linear genome maps, density plots, and heatmaps using matplotlib/seaborn, enabling rapid inspection of motif frequency, position, and diversity. Core dependencies are MEME Suite, Biopython, SeqKit, R (ggplot2/ggtree ecosystem for logos in the helper script), pandas, and Python's logging/CLI. Inputs are FASTA files (DNA or protein) plus optional metadata; outputs are structured TSV/CSV tables, MEME XML/EPS, split FASTAs, and figures. Default parameters (e.g., -nmotifs 1, -minw 5, -maxw 10) target the short (5–10 aa/nt) signatures; all thresholds are intentionally exposed so users can broaden searches (more motifs, wider windows) or tighten detections (exact regex, no-gap scans) depending on dataset size and hypothesis.

S1.5 Putative Stem Loop and Iterons Annotation

The putative stem-loop and iteron annotation suite integrates secondary-structure prediction with repeat-pattern mining to localize replication origins in CRESS-DNA genomes. The stem-loop component scans user genomes in FASTA format near a conserved nonanucleotide that can be provided directly through the motif option or inferred from a selected viral family, folding flanking windows defined by the frame parameter to identify hairpins whose helix and loop dimensions approximate canonical origins specified by idealstemlen and ideallooplen. Candidate loci receive scores that penalize departures from the target geometry and are adjusted for motif concordance, after which they are written as GFF3 features named stem_loop and nonanucleotide with an optional tabular summary that records motif sequence, coordinates, hairpin score, and minimum-free-energy structure. This step employs ViennaRNA for folding and dot-bracket structures through its RNA interface together with Biopython for FASTA input and gffutils for lightweight parsing and emission of GFF. The intended inputs comprise a genome FASTA and a GFF providing at least a region definition, while the outputs comprise a GFF3 annotation set and an optional CSV that support origin-of-replication curation, cross-family comparison, and seeding of downstream iteron searches. The principal parameters are motif or family for seeding, frame for the folding window, idealstemlen and ideallooplen for the target geometry, and file path options for the incoming and outgoing records.

The iteron detector then restricts its analysis to neighborhoods anchored by the annotated hairpin and nonanucleotide and enumerates short tandem repeats within a buffered window controlled by the range parameter, ranking candidates by spacing regularity using wiggle, bestDist, and maxDist together with penalties for low-complexity composition and with length heuristics defined by minLength, maxLength, and goodLength. Users may retain the highest-ranked set using rank, numberTopIterons, and scoreRange or filter by an absolute score

threshold using maxScore and optionally preserve repeats that overlap the stem loop with doStemLoop or tag known motifs with doKnownIterons. For each genome the workflow creates per-contig intermediate GFFs, evaluates iterons, and merges results into a final GFF3 that contains iteron and when applicable stem_loop_repeats features, while recording execution statistics in a log and cleaning transient directories on exit. The required inputs consist of the genome FASTA and the GFF produced by the stem-loop step along with the user parameters governing search scale and stringency, and the outputs consist of a consolidated GFF3 suitable for genome-browser inspection or downstream rule-based filtering together with logs.

S2. Building CRESSENT Rep and Cap Proteins Database for Phylogenetic Analysis

Building the CRESSENT database (**Fig. S1A**) begins with the download of family-level protein sequences retrieved using the NCBI Entrez API based on the most recent Master Species List (MSL) from ICTV (https://ictv.global/msl/current) filtered by only ssDNA virus accession numbers. Then, CD-HIT was employed to cluster sequences at 95% amino acid identity with a minimum 90% coverage of the shortest sequence, using parameters -c 0.95 -aS 0.9 -M 16000 -T 8, followed by all-vs-all sequence comparison using Diamond BLASTP with an e-value threshold of 1e-5 and parameters --masking 0 --sensitive. The Markov Clustering (MCL) algorithm was used with an inflation rate of 1.5 on the Diamond BLASTP results (query, subject, and e-value columns), generating protein family clusters. Finally, sequences were assigned with protein descriptions to each cluster, creating comprehensive tables linking clusters to their sequences and annotations, followed by functional classification: capsid proteins (containing terms such as “cap,” “Capsid,” or “VP” with at least 8 proteins per cluster), replication proteins (terms such as “rep,” “Rep,” or “replicase” with at least 1 protein per cluster), and unannotated ORFs (clusters not classified as capsid or rep, with at least 10 proteins per cluster). Separate directories and files are generated for each category using custom R and Python scripts.

After building the database, we aimed to evaluate the similarity among the Capsid (Cap) and Replication (Rep) protein sequences using the similarity networks (SSNs) algorithm (**Fig. S1 B-C**). For this, sequences from each dataset were concatenated and submitted to the EFI-EST [1] platform for SSN generation, and the resulting networks were visualized using Cytoscape [2]. Specifically for Rep domains, sequences were first grouped by viral family and aligned using the align module from CRESSENT. Sequences were analyzed to pinpoint the position of the Walker A motif within each family. Based on the identified motif positions, regular expressions (regex) were created using a custom script. These regex patterns were subsequently used with the motif module to split each family’s sequences into functional domains (HUH and S3F domains). The extracted domain sequences were then concatenated and analyzed in EFI-EST to produce domain-specific SSNs (**Fig. S1 D-E**), which were again visualized in Cytoscape. Networks at a 95% similarity threshold were examined, and associated metadata were used to annotate and color nodes by viral family, facilitating comparative analysis across groups.

Cap protein sequences (**Fig. S1 B**) exhibited more cohesive clustering overall compared to Rep proteins (**Fig. S1 C**). However, when analyses focused on individual domains (HUH and S3F), Rep proteins formed clearer, more distinct clusters (**Fig. S1 D-E**). Therefore, users should exercise caution when using full-length Rep sequences from diverse ssDNA virus families in a

single analysis. We recommend conducting domain-level analysis and treating each Rep domain separately for accurate comparative studies.

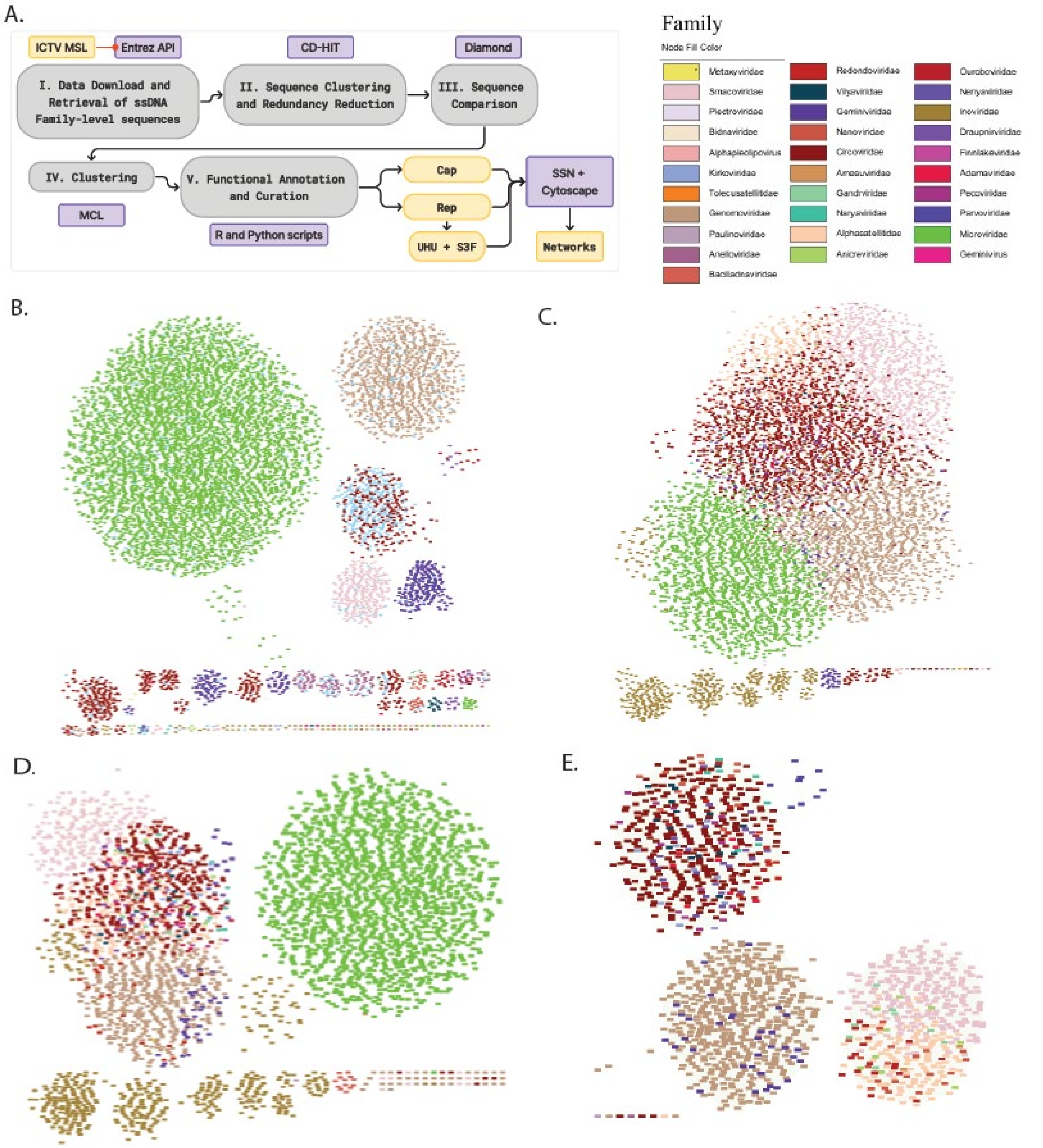

Fig S1: CRESSENT Database. (A) Workflow used to build the custom database of CRESSENT. gray boxes: workflow steps; purple boxes: tools or scripts; yellow boxes: input/output files. Protein sequence similarity network (SSN) of (B) Cap, (C) Rep proteins, (D) HUH, and (E) S3F domains. The SSNs were generated using EFI-EST. Sequences sharing 95% identity are conflated as a

single node and visualized by Cytoscape. Nodes are colored by family (see legend on upright hand side).

S3. Capabilities

Sequences from two independent studies were employed to demonstrate the utility of CRESSENT in facilitating the annotation of Rep and Caps genes in two putative viral families, *Naryaviridae* [3] (**Fig. S2 and Fig. S3**) and *Genomoviridae* [4] **(Fig. S4)**. These viral sequences were identified and annotated using Cenote-Taker3 after undergoing quality trimming, assembly, and clustering. For more details, you can access our documentation: https://cressent.readthedocs.io/en/latest/

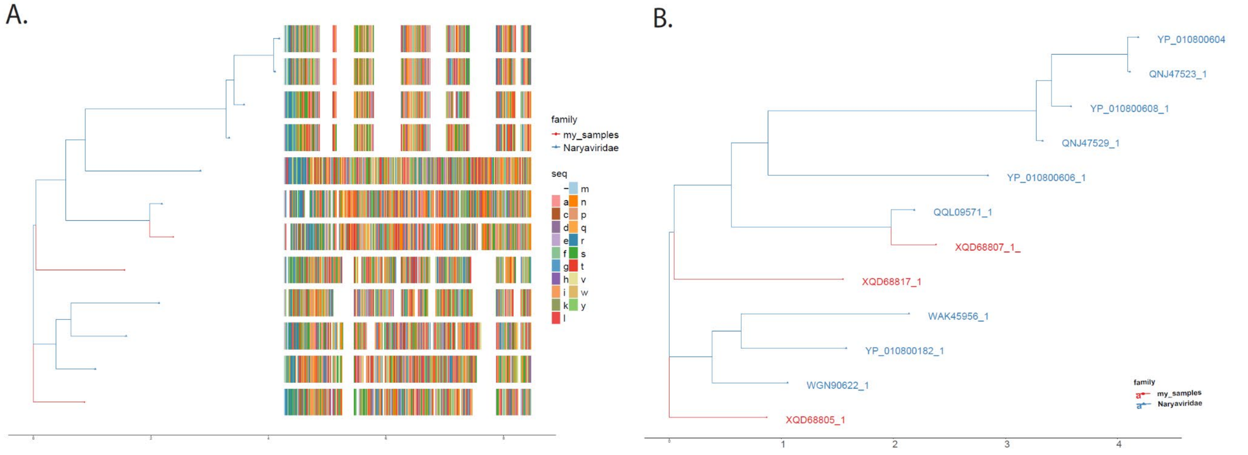

Fig S2 - Tree visualization by CRESSENT: Phylogenetic trees of Cap proteins (red = study samples; blue = custom DB sequences) produced by CRESSENT. Phylogenetic trees (A) with (B) and without alignment.

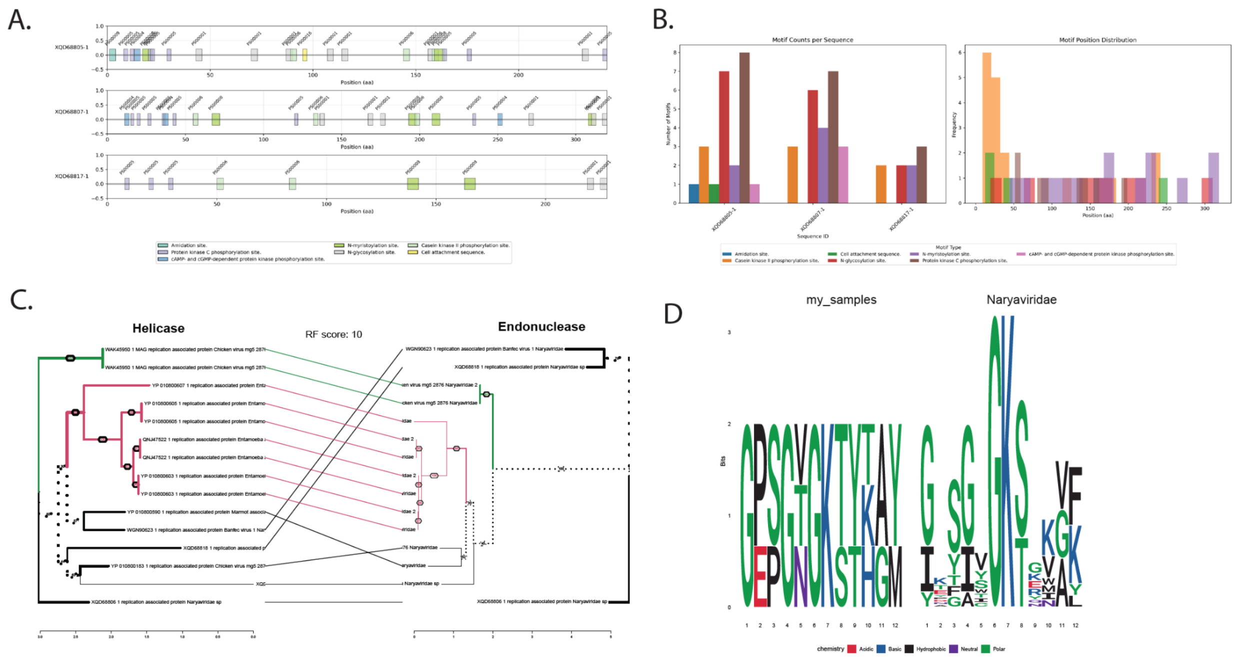

Fig S3 - Visualizations of CRESSENT modules: (A and B) Gene map visualization of the motifs found in the study sequences [3]) with the *motif_disc* module. (C) Tanglegram of the Rep proteins and (D) sequence logo of the Walker A motif of Rep proteins split by study and database sequences.

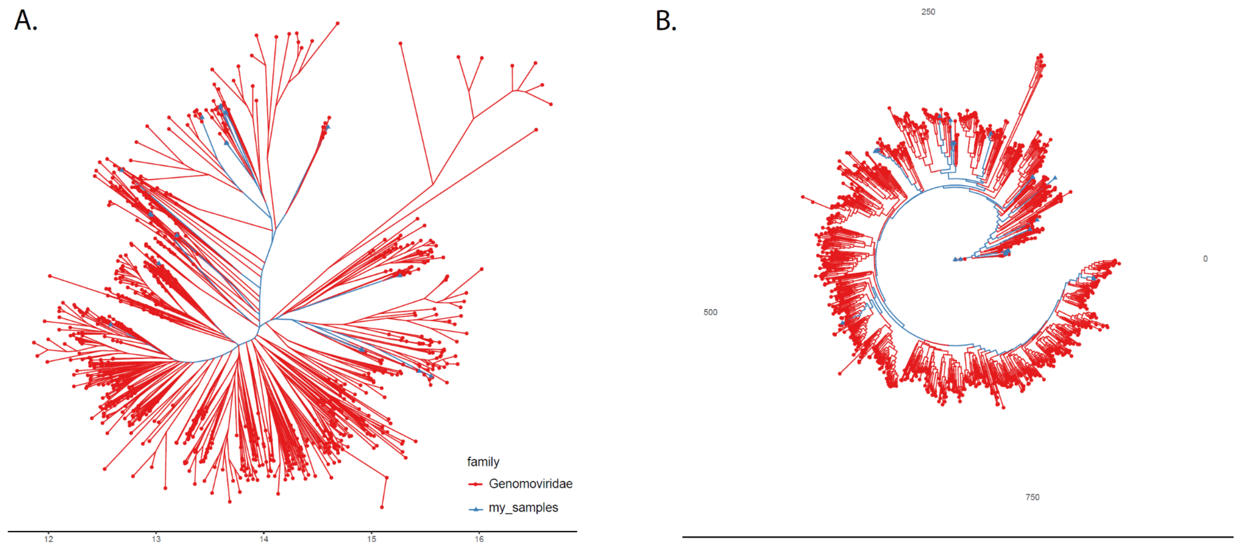

Fig S4 - CRESSENT allows different tree visualizations: Unrooted (B) and circular (A) phylogenetic trees of the *Genomoviridae* Cap genes (blue = study samples [4]; red = custom DB sequences).

S4. REFERENCES

1. Oberg N, Zallot R, Gerlt JA. EFI-EST, EFI-GNT, and EFI-CGFP: Enzyme Function Initiative (EFI) Web Resource for Genomic Enzymology Tools. J Mol Biol [Internet]. 2023 Jul 15 [cited 2025 Jun 11];435(14):168018. Available from: https://www.sciencedirect.com/science/article/pii/S0022283623000748

2. Shannon P, Markiel A, Ozier O, Baliga NS, Wang JT, Ramage D, et al. Cytoscape: A Software Environment for Integrated Models of Biomolecular Interaction Networks. Genome Res [Internet]. 2003 Nov 1 [cited 2025 Jun 11];13(11):2498–504. Available from: http://genome.cshlp.org/content/13/11/2498

3. Zhang H, Fu Y, Cao C, Jiang H, Tang R, Dai Z, et al. Identification and Characterization of Novel CRESS-DNA viruses in the Human Respiratory Tract [Internet]. Research Square; 2025 [cited 2025 Jun 23]. Available from: https://www.researchsquare.com/article/rs-6208723/v1

4. Leal Rodríguez C, Shah SA, Rasmussen MA, Thorsen J, Boulund U, Pedersen CET, et al. The infant gut virome is associated with preschool asthma risk independently of bacteria. Nat Med [Internet]. 2024 Jan [cited 2024 Mar 28];30(1):138–48. Available from: https://www.nature.com/articles/s41591-023-02685-x
